# Angiotensin II Type 1 Receptor Antibody-Mediated Kidney Rejection Unresponsive to Treatment

**DOI:** 10.7759/cureus.41007

**Published:** 2023-06-26

**Authors:** David Allison, Zahraa Hajjiri, Luis Manon, Sally Campbell-Lee, Suhalika Sahni, Suman Setty

**Affiliations:** 1 Pathology, University of Illinois at Chicago, Chicago, USA; 2 Transplant Nephrology, University of Illinois at Chicago, Chicago, USA

**Keywords:** renal transplant rejection, apheresis therapy, at1r antibody, non-hla antibody, kidney allograft, therapeutic plasma exchange (tpe)

## Abstract

Allograft rejection is a significant cause of renal transplant failure which needs prompt diagnosis and treatment for graft salvage. Angiotensin II type 1 receptor antibody-mediated rejection (AT1R-AMR) is increasingly being identified as the etiology of antibody-mediated rejection in kidney transplant recipients with allograft rejection but without detectable human leukocyte antigen (HLA) antibodies. While some reports have suggested that AT1R-AMR may be refractory to standard therapy, others have reported improvement or stabilization of graft function. We present two patients in which anti-rejection therapy including therapeutic plasma exchange was unable to salvage the allograft.

## Introduction

Kidney transplant is the treatment of choice for end-stage renal disease (ESRD), with well-documented survival rates superior to patients maintained on dialysis. This survival advantage is most pronounced in younger patients with diabetes mellitus, with post-transplant survival of 25 years versus eight years on dialysis [1]. Unfortunately, the number of patients awaiting an organ far outpaces the number of transplants performed (100,000 patients versus 19,000 transplants in 2016), and the average wait time ranges between three and a half to six years [1,2]. Multiple factors influence a patient’s length of wait, including type of kidney donor (deceased versus living donor) and blood type. Blood groups O and B tend to have longer waits than groups A and AB, and half or more of patients waiting for an organ are group O [3]. One single-center retrospective study demonstrated that group O recipients waited significantly longer than non-O recipients for a deceased donor kidney (mean 79.6 months versus 57.5 months) [4].

Rejection is a well-known complication following kidney transplant, resulting in graft failure if not treated promptly. Rejection is the recipient’s immune response against the kidney allograft and can be either cellular (T cell-mediated rejection [TCMR]) or humoral (antibody-mediated rejection [AMR]). TCMR occurs more frequently, but AMR is involved in between 12-37% of acute rejection [2]. Cellular and humoral rejection may occur together in the same patient, demonstrable in allograft biopsies. Most AMR cases are due to recipient antibodies against donor human leukocyte antigen (HLA) antigens. Recently, non-HLA antibodies have been identified as causing AMR in the absence of recipient HLA sensitization. Among these, antibodies with agonistic specificity for the angiotensin II type 1 receptor (AT1R) are increasingly implicated in cases of AMR refractory to standard treatment [5,6].

Institutional review board (IRB) exemption was granted (STUDY2023-0191) to conduct a retrospective two-patient case series. This brief report presents two patients with AT1R-AMR treated with therapeutic plasma exchange (TPE) and intravenous immunoglobulin, and their outcomes. In both cases, the clinical diagnosis of rejection is correlated with renal allograft biopsies. A brief literature review and summary table of previously reported cases are included. This article was previously presented as a meeting poster at the 2023 Midwest Clinical and Translational Research Meeting on April 24, 2023.

## Case presentation

Patient 1

A 53-year-old woman with a history of ESRD due to hypertension, status post living related renal transplant (LRRT) three years ago was admitted with an elevated serum creatinine (Cr) of 3.4 mg/dL (baseline 1 - 1.2 mg/dL). She received a haploidentical kidney from her daughter. At the time of transplant, the HLA antibody screen was negative with a negative crossmatch. Her maintenance immunosuppression was tacrolimus and mycophenolic acid. Her admission work-up revealed subtherapeutic tacrolimus levels.

**Figure 1:**
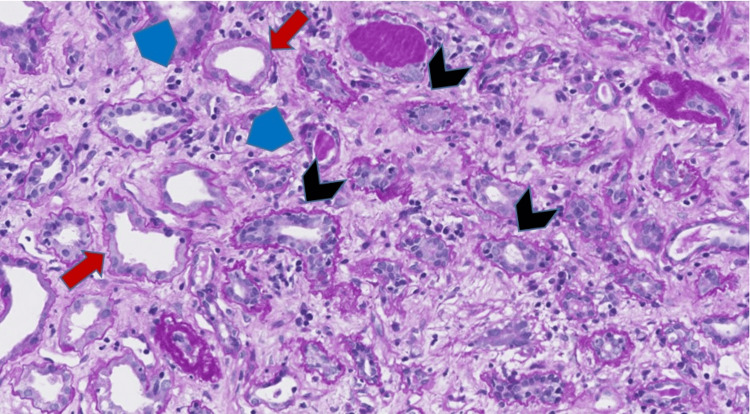
Allograft biopsy, patient 1 Tubulitis (black chevrons) and interstitial inflammation consistent with T cell-mediated rejection (TCMR). Tubular luminal dilatation with attenuation of brush borders (red arrows) and peritubular capillaritis (blue pentagons) (Periodic-Acid Schiff stain, original magnification 200x).

**Figure 2:**
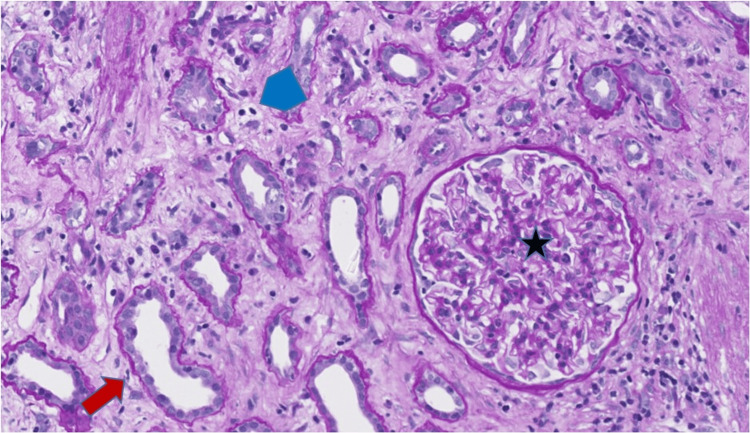
Allograft biopsy, patient 1 Tubular luminal dilatation with attenuation of brush borders (red arrow), glomerulitis (star), and peritubular capillaritis (blue pentagon) (Periodic-Acid Schiff stain, original magnification 200x).

During hospitalization, she underwent a kidney allograft core biopsy that demonstrated tubulitis and interstitial lymphocytic inflammation, changes of acute TCMR, Banff 1A (Figures 1, 2). Glomerulitis, peritubular capillaritis and acute tubular necrosis were present, consistent with concomitant AMR. C4d was focally positive in the peritubular capillaries as seen by immunohistochemistry. She was administered intravenous methylprednisolone 500 mg for three days for TCMR, then continued on oral prednisone 10 mg daily.

Donor-specific antibodies (DSA) were not detected, and AT1R antibody assay was sent to a reference laboratory. A right internal jugular vein double lumen tunneled dialysis catheter was inserted in preparation for an initial series of five to seven TPE followed by intravenous immunoglobulin (IVIgG) 10 g, every other day. TPE was performed with the Spectra Optia (TerumoBCT, Lakewood, CO, USA), using human albumin 5% as the replacement fluid and anticoagulant citrate dextrose solution A (ACD-A). She underwent two TPE of one plasma volume (PV) each during admission and was discharged on hospital day 11 with creatinine 3.06 mg/dL.

Ten days after discharge, the AT1R antibody result was received from the reference lab, as “positive” at 27.3 units/mL (reference range: <10 units/mL is negative; 10 - 17 units/mL is borderline; >17 units/mL is positive). TPE was re-initiated as an outpatient three times a week, for three weeks in the setting of non-HLA AMR. All procedures were performed with the Spectra Optia, exchanging one PV with albumin 5%, using ACD-A. This treatment course was complicated and interrupted by multiple brief hospitalizations for suspected community-acquired pneumonia and pulmonary edema. Following a course of antibiotics and diuresis, the TPE schedule was resumed, although IVIgG was discontinued due to the risk of volume overload. Losartan was also added. Her AT1R antibody assay was repeated after a prolonged series of outpatient procedures, resulting as “negative” at 4.3 units/mL, although her Cr had increased.

She underwent 26 TPE sessions in total, with progressively increasing serum Cr, which had risen to 8.3 mg/dL by the end of the course of TPE. Unfortunately, the transplant failed, and peritoneal dialysis was restarted.

Patient 2

**Figure 3:**
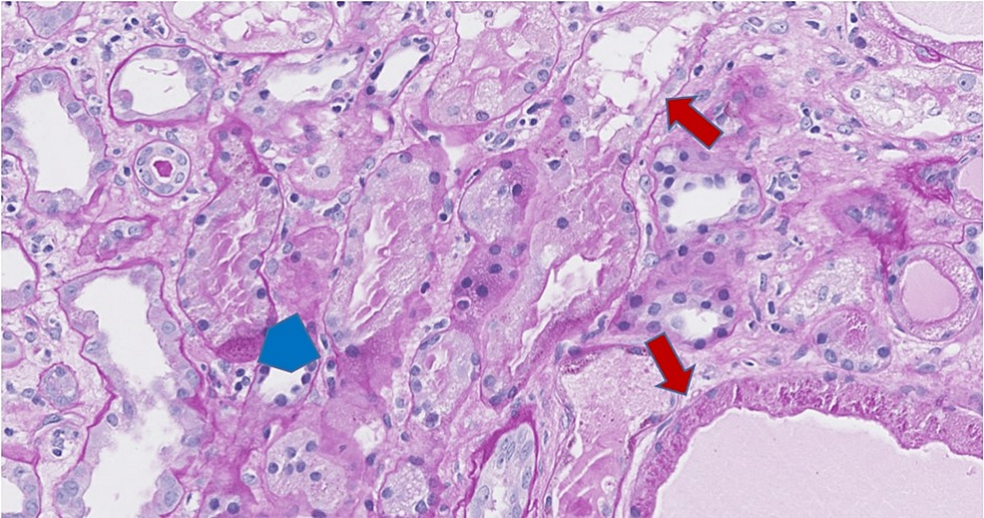
Allograft biopsy, patient 2 Tubular luminal dilatation with attenuation of brush borders (red arrows) and peritubular capillaritis (blue pentagon) (Periodic-Acid Schiff stain, original magnification 200x).

**Figure 4:**
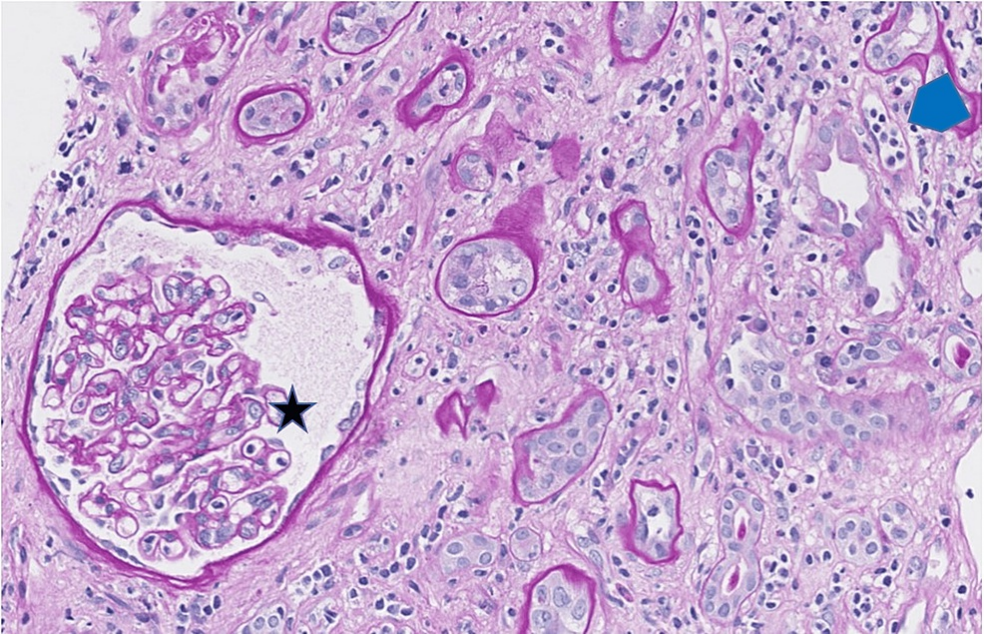
Allograft biopsy, patient 2 Glomerulitis (star) and peritubular capillaritis (blue pentagon) (Periodic-Acid Schiff stain, original magnification 200x).

A 62-year-old male with history of ESRD status post deceased donor renal transplant (DDRT) two years ago presented with proteinuria. He had never demonstrated any DSA, yet he developed increasing proteinuria of 1,109 mg/dL (urine protein/creatinine ratio 8.21) and rising serum Cr to 2.74 mg/dL (baseline Cr 2 mg/dL), while on therapeutic doses of tacrolimus, along with prednisone 5 mg daily and mycophenolic acid 720 mg twice daily. The kidney allograft core biopsy (Figures 3, 4) showed changes of acute tubular necrosis, glomerulitis and peritubular capillaritis and was negative for C4d by immunohistochemistry, suspicious for AMR.

AT1R antibody was sent to a reference laboratory and returned as “positive” >40 units/mL. The patient was started on losartan 25 mg, but his serum Cr continued to rise to 3.02 mg/dL. Losartan was discontinued, and the patient was scheduled for a series of six TPE treatments, every other day for two weeks, each followed by IVIgG infusion. TPE was performed with the Spectra Optia using human albumin 5% as the replacement fluid and ACD-A. One PV was exchanged during each procedure.

He underwent six sessions of TPE in total, with overall worsening of serum Cr (2.97 mg/dL). His renal function further declined and he restarted hemodialysis three months later.

## Discussion

Accurate, rapid diagnosis of rejection is necessary to preserve organ function. Allograft biopsy can help differentiate TCMR and AMR, which guides appropriate treatment. Two important points to remember about biopsies are that TCMR and AMR may coexist in one patient, and negativity for C4d (either by immunohistochemical stain or immunofluorescence) does not exclude AMR [7]. Identifying DSAs or other anti-HLA antibodies also supports an AMR diagnosis, but their absence does not rule AMR out entirely. While antibodies against HLA antigens are the primary cause of antibody-mediated rejection in renal transplant, clinically significant non-HLA antibodies are now being described, particularly in recipients serologically negative for HLA antibodies who exhibit signs of AMR. Examples of implicated non-HLA antibodies include anti-endothelial cell antibodies (anti-EC), anti-major histocompatibility complex class I chain-related gene A antibodies (anti-MICA), and AT1R antibodies. Preformed AT1R antibodies have been shown to independently influence renal transplantation success [6,8]. These antibodies exert an agonist effect upon the angiotensin II type 1 receptor, increasing vasoconstriction, pro-inflammatory mediators, and thrombosis. At least some of these cases are complement-independent, evidenced by C4d non-reactivity in many renal biopsies [8]. Such was the case in our two patients. AT1R antibodies are of interest owing to their unique mechanism of action (and potential pharmacologic target) and availability of a commercial assay. The exact relationship between AT1R antibodies and graft failure has not been established but may include vasoconstriction or thrombotic occlusion causing vascular rejection [5]. However, enough literature is present to indicate that the role of these antibodies adds to the patients’ immunological risk profile, putting them at risk for graft failure [6]. Therefore, in cases of graft dysfunction with negative DSA, AT1R antibodies should be considered and tested for.

The optimal therapy for AT1R-AMR is an active area for research, given the literature suggests that these patients may be refractory to standard treatment regimens based on a novel mechanism of injury compared to HLA antibodies. Angiotensin receptor blockers (ARBs), a class of anti-hypertensive medications, are often trialed in these patients. Angiotensin receptor blockade is thought to inhibit the agonist effect of AT1R antibodies. Previous case reports have documented improvement in allograft function following TPE and IVIgG (Table 1). However, there could be yet-unidentified antibodies against the donor kidney in addition to other patient-specific factors that may explain a poor therapeutic response in some cases but recovery in others. Both of our cases had unfortunate outcomes of graft failure, despite standard anti-rejection treatment that included TPE.

**Table 1:** Summary of Cases Treated with TPE Abbreviations: TPE, therapeutic plasma exchange; IVIgG, intravenous immunoglobulin; ARB, angiotensin receptor blocker; NR, not reported

	No. of cases	TPE sessions	IVIgG	Other immunosuppression	ARB use?	Outcome
Dragun [5]	7	Yes (NR)	Yes	NR	Yes	Graft salvage
Yamada [9]	2	8 – 11	Yes	NR	Yes	Graft salvage
Wiwattanathum [10]	1	11	Yes	Methylprednisolone, anti-thymocyte globulin, tacrolimus, rituximab	No	Graft salvage
Jobert [11]	1	6	No	Methylprednisolone, anti-thymocyte globulin	Yes	Graft salvage
Fuss [12]	11	6 - 11	Yes	Methylprednisolone, anti-thymocyte globulin	Yes	Graft salvage
Lee [13]	10	Yes (NR)	Yes	Steroids (unspecified), anti-thymocyte globulin, rituximab, bortezomib (1 case)	NR	Graft salvage
Abuzeineh [14]	8	Yes (NR)	Yes	Steroids, rituximab	Yes	Graft salvage in 3 of 8
Our report	2	26 (case 1) 6 (case 2)	Yes	Methylprednisolone, tacrolimus	Yes	Graft failure

## Conclusions

Renal transplantation is the treatment of choice for ESRD, but donor organs are scarce. Prompt diagnosis and treatment of AMR is necessary to preserve organ function, and most cases of AMR are due to anti-HLA antibodies. However, in situations where anti-HLA antibodies and DSAs are not identified, the clinician needs to consider AT1R-AMR. Although a rare cause of transplant rejection, AT1R-AMR may need more aggressive therapy.
